# Chromosomal distribution of microsatellite repeats in Amazon cichlids genome (Pisces, Cichlidae)

**DOI:** 10.3897/CompCytogen.v9i4.5582

**Published:** 2015-09-14

**Authors:** Carlos Henrique Schneider, Maria Claudia Gross, Maria Leandra Terencio, Édika Sabrina Girão Mitozo de Tavares, Cesar Martins, Eliana Feldberg

**Affiliations:** 1 Universidade Federal do Amazonas, Instituto de Ciências Biológicas, Departamento de Genética, Laboratório de Citogenômica Animal, Av. General Rodrigo Otávio, 3000, Japiim, Zip code 69077-000 Manaus, AM, Brazil; 2 Universidade Federal da Integração Latino Americana, Laboratório de Genética, Av. Tarquínio Joslin dos Santos, 1000, Jardim Universitário, Zip code 85857-190, Foz do Iguaçu, PR, Brazil; 3 Universidade Estadual Paulista Júlio de Mesquita Filho – UNESP, Instituto de Biociências, Departamento de Morfologia, Laboratório Genômica Integrativa, Rubião Junior, Zip code 18618-000 Botucatu, SP, Brazil; 4 Instituto Nacional de Pesquisas da Amazônia, Laboratório de Genética Animal, Av. André Araújo, 2936 Zip Code 69077-000, Manaus, AM, Brazil

**Keywords:** Karyotype evolution, fluorescence *in situ* hybridization, repetitive DNA, genome organization

## Abstract

Fish of the family Cichlidae are recognized as an excellent model for evolutionary studies because of their morphological and behavioral adaptations to a wide diversity of explored ecological niches. In addition, the family has a dynamic genome with variable structure, composition and karyotype organization. Microsatellites represent the most dynamic genomic component and a better understanding of their organization may help clarify the role of repetitive DNA elements in the mechanisms of chromosomal evolution. Thus, in this study, microsatellite sequences were mapped in the chromosomes of *Cichla monoculus* Agassiz, 1831, *Pterophyllum scalare* Schultze, 1823, and *Symphysodon discus* Heckel, 1840. Four microsatellites demonstrated positive results in the genome of *Cichla monoculus* and *Symphysodon discus*, and five demonstrated positive results in the genome of *Pterophyllum scalare*. In most cases, the microsatellite was dispersed in the chromosome with conspicuous markings in the centromeric or telomeric regions, which suggests that sequences contribute to chromosome structure and may have played a role in the evolution of this fish family. The comparative genome mapping data presented here provide novel information on the structure and organization of the repetitive DNA region of the cichlid genome and contribute to a better understanding of this fish family’s genome.

## Introduction

The fish family Cichlidae exhibits high species richness with approximately 3,000 species distributed in Central and South America, Africa, and South India (Kullander 1998, Kocher 2004). The evolution of this family is characterized by repeated adaptive radiation and sympatric speciation (Schliewen et al. 1994, Seehausen 2006). Moreover, these fish are considered to be an excellent model for evolutionary studies because of their morphological and behavioral adaptations to a wide diversity of explored ecological niches (Lowe-McConnell 1991). Approximately 400 species have been identified in a wide range of habitats in the Neotropical region. The Amazon exhibits the highest diversity of Cichlidae, with more than 300 identified species (Kullander 2003).

In Neotropical cichlids, the diversity of morphological adaptations does not result from variations in the diploid number because most species have 48 chromosomes (Feldberg et al. 2003). However, the species of this family exhibit a dynamic genome with variations in structure and karyotype composition and organization, as demonstrated by the DNA sequencing and the physical chromosome mapping of several repetitive DNA sequences, such as telomere sequences; retrotransposons isolated from *Xiphophorus maculatus* Günther, 1866 (*Rex*1, *Rex*3, *Rex*6); retrotransposon isolated from *Astronotus ocellatus* Agassiz, 1831 similar to *Rex*3 (Ao*Rex*3); long interspersed elements isolated from *Astronotus ocellatus* (AoLINE); retrotransposon isolated from *Cichla kelberi* Kullander & Ferreira, 2006 (RCk); transposon isolated from *Caenorhabditis elegans* Maupas, 1900 (*Tc*1); the 18S and 5S ribosomal gene sequences; and U1 spliceosomal small nuclear RNA (U1 snRNAs) (Vicari et al. 2006, Gross et al. 2009, Mazzuchelli and Martins 2009, Teixeira et al. 2009, Gross et al. 2010, Poletto et al. 2010, Cabral-de-Mello et al. 2012, Valente et al. 2011, Schneider et al. 2013a, Schneider et al. 2013b). Moreover, in this fish family, repetitive DNAs, such as transposable elements, co-localize or are associated with ribosomal DNAs, which suggests their roles in the duplication and dispersion of repetitive rDNA sequences (Gross et al. 2010, Schneider et al. 2013a, Schneider et al. 2013b, Nakajima et al. 2012).

Repetitive DNA sequences display a high degree of polymorphism because of the variation in the number of repetitive units, which results from a specific evolutionary dynamics. Among these elements, microsatellites (or short tandem repeats) are the most polymorphic and are formed of short sequences of one to six nucleotides repeated in tandem throughout the DNA (Tautz and Renz 1984). Because of their supposed neutral evolution, these molecular markers have been widely used in population genetics, to identify taxonomic limits, and in hybridization and forensic studies (Goldstein and Pollock 1997, Filcek et al. 2005, Racey et al. 2007, McCusker et al. 2008). However, recent research has demonstrated that certain microsatellites are functional and may affect gene regulation (Kashi and King 2006, Gemayel et al. 2010, Sonay et al. 2015); evolution of orphan genes (Palmieri et al. 2014, Schlötterer 2015); involved in chromosomal rearrangements (Kamali et al. 2011) and be involved with increased or diminishes likelihood of disease related alleles size (Ananda et al. 2014; Padeken et al. 2015).

The chromosomal mapping of microsatellite sequences has been little examined. This approach is primarily used to study the evolution of different sex-determining chromosome systems (Li et al. 2010, Pokorná et al. 2011, Terencio et al. 2013). Considering that microsatellites are the most dynamic genomic component, a better understanding of their chromosomal organization is important for improving knowledge regarding the role of repetitive DNA elements in the mechanisms of chromosomal evolution and heterochromatin composition.

*Cichla monoculus* Agassiz, 1831 has a karyotype with 2n = 48 subtelo/acrocentric (st/a) chromosomes, described as basal for cichlids, and little heterochromatin. Although *Pterophyllum scalare* Schultze, 1823 also has 2n = 48 chromosomes, this species differs in karyotype formula with meta/submetacentric (m/sm) chromosomes due to chromosomal inversions and accumulation of heterochromatin in the pericentromeric regions. The highest diploid number described for this group is found in species of the genus *Symphysodon* Heckel, 1840, which has 2n = 60 chromosomes, as well as large heterochromatic blocks (Schneider et al. 2013a). Thus, this study provides a physical mapping of microsatellite sequences on the chromosomes of three Neotropical cichlid fish species (*Cichla monoculus*, *Pterophyllum scalare*, and *Symphysodon discus*), that occupy different phylogenetic positions, and contributes to a better understanding of the chromosomal organization and evolution of this fish family.

## Methods

Specimens of *Cichla monoculus* (four males and four females), *Pterophyllum scalare* (three males and three females) were collected in Catalão Lake, confluence of the Negro/Solimões Rivers (3°09'47.44"S / 59°54'51.39"W) and *Symphysodon discus* (two males and two females) in Negro River (0°56'06.43"S / 62°56'22.61"W). The specimens were caught in the wild with sampling permission (ICMBio SISBIO 10609-1/2007). All of the individuals were euthanatized with Eugenol (clove oil).

Mitotic chromosomes were obtained from kidney cells using an air-drying protocol (Bertollo et al. 1978).

Eight microsatellites (Amado et al. 2008; Passos et al. 2010) were mapped using fluorescence *in situ* hybridization (FISH) during the mitotic metaphase of *Cichla monoculus*, *Pterophyllum scalare*, and *Symphysodon discus* (Table 1). The repetitive sequence probes were labeled with digoxigenin-11-dUTP or biotin-16-dUTP (Dig-Nick Translation Mix and BioNick Translation Mix; Roche) using nick translation reactions following the manufacturer’s instructions. Anti-digoxigenin rhodamine (Roche) and streptavidin/Alexa Fluor 488 (Life Technologies) were used to detect the signal. FISH was performed on mitotic chromosome spreads (Pinkel et al. 1986). The FISH was performed with high stringency (2.5 ng/µl of DNA, 50% deionized formamide, 10% dextran sulfate and 2xSSC at 37 °C for 18 h). The chromosomes were counterstained with DAPI (2 µg/ml) in the Vectashield mounting medium (Vector).

**Table 1.** Repetitive sequences hibridized to cichlid chromosomes. (+) positive hybridization signals detected; (-) absence of hibridization signals.

Repeat motif	*Cichla monoculus*	*Pterophyllum scalare*	*Symphysodon discus*
(CA)_16_	+	+	+
(AC)_7_	+	-	-
(GT)_13_	+	+	-
(GA)_12_	-	+	-
(GAATA)_8_	+	+	+
(GAGAA)_12_	-	+	-
(GT)_9_CA(GT)_7_CG(GT)_19_	-	-	+
(CT)_14_GT(CT)_5_(CG)_2_(CT)_9_	-	-	+

## Results

**Figure 1.**
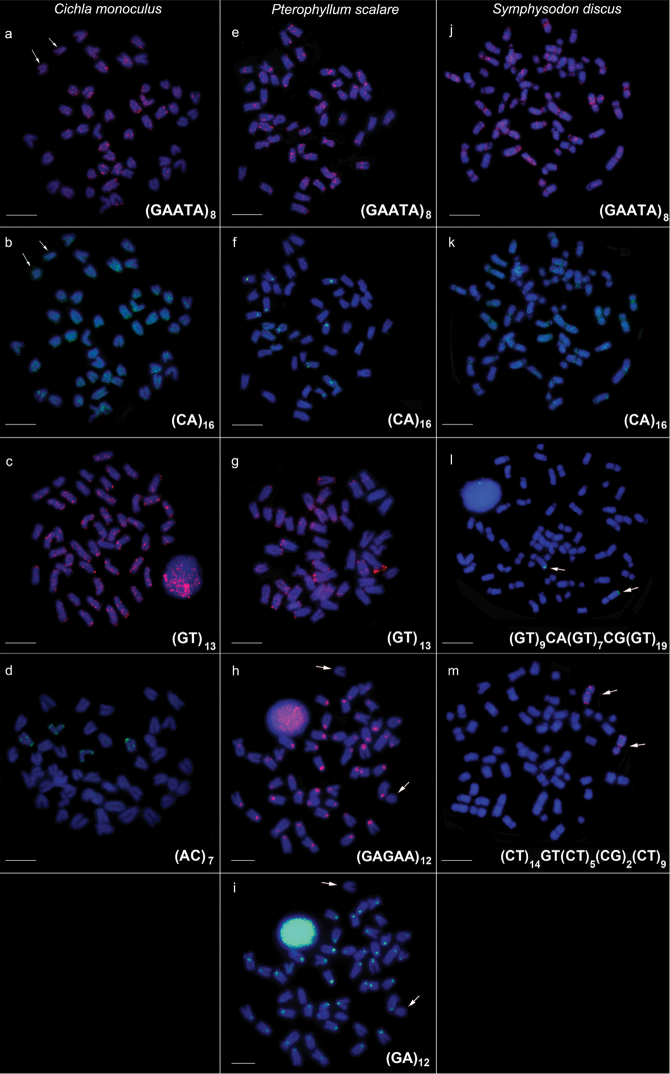
Metaphase chromosomes of *Cichla monoculus*, *Pterophyllum scalare* and *Symphysodon discus* hybridized with microsatellite sequences (**a–m**). Arrow shows no signs of hybridizations in **a, b, h, i** and chromosomes positive for microsatellite in **l, m**. The probes detection was performed with streptavidin Alexa Fluor 488 (green) or anti-digoxigenin rhodamine (red). Chromosomes were counterstained with DAPI. Scale bar: 10 µm.

Four microsatellites, among which three were dinucleotides and one was a pentanucleotide, exhibited positive reactions in the genome of *Cichla monoculus* (Table 1). Hybridizations with the pentanucleotide microsatellite (GAATA)_8_ displayed dispersed signals in all of the chromosomes. Moreover, conspicuous markings were observed in several chromosome pairs. However, one chromosome pair did not exhibit any homology with the probe (Fig. 1a). The microsatellite (CA)_16_ was distributed in all of the chromosomes of *Cichla monoculus*, except for one pair. In most chromosomes, the microsatellite displayed a dispersed distribution, and in several cases, the markings were conspicuous (Fig. 1b). A dispersed pattern was observed after hybridization with the microsatellite (GT)_13_, whereas a strong signal occurred in the telomeric, interstitial or centromeric regions of the chromosomes (Fig. 1c). Conversely, hybridizations with the microsatellite (AC)_7_ resulted in only two positive chromosome pairs, one with markings in the telomeric region of the short arm and in the interstitial region of the long arm and the other with markings in both telomeric regions (Fig. 1d).

Five microsatellites were mapped in the genome of *Pterophyllum scalare*: three dinucleotides and two pentanucleotides (Table 1). The microsatellites (GAATA)_8_, (CA)_16_ and (GT)_13_, which were dispersed in *Cichla monoculus*, exhibited clustered signals in *Pterophyllum scalare*. The microsatellite (CA)_16_ was mapped on centromeric blocks of five chromosome pairs (Fig. 1f), other signals were located in telomeric, centromeric and interstitial regions of most chromosomes (Fig. 1e and 1g). Additionally, the microsatellites (GA)_12_ and (GAGAA)_12_ exhibited similar patterns of conspicuous markings in the centromeres. However, in the case of (GAGAA)_12_, one chromosome pair did not display any markings (Fig. 1h, i).

Four microsatellites were mapped in *Symphysodon discus*. In this species, the microsatellites (GAATA)_8_ and (CA)_16_ exhibited patterns similar to that of *Cichla monoculus* with dispersed signals and conspicuous markings in the centromeric region (Fig. 1j, 1k). Only one chromosome pair displayed positive results for microsatellites (GT)_9_CA(GT)_7_CG(GT)_19_ and (CT)_14_GT(CT)_5_(CG)_2_(CT)_9_. The first was located in the telomeric region of the long arm, and the latter was located in the centromeric and telomeric regions (Fig. 1l, m).

## Discussion

The repetitive regions of the genome typically do not undergo the selective pressure that affects non-repetitive sequences, and most microsatellite sequences evolve neutrally and supposedly do not affect an individual phenotype (Schlötterer 2000). However, recent studies have identified functional microsatellites that affect the physical aspect of an individual (Kashi and King 2006, Gemayel et al. 2010, Padeken et al. 2015). These putative functional microsatellites are primarily located in or near gene regions, and there is variation in the number of times that the motif is repeated, which is related to the ability of the microsatellites to modify gene expression or change protein sequences (Wren et al. 2000, Li et al. 2004, Vinces et al. 2009, Gemayel et al. 2010). In addition to this functional aspect, repetitive DNA variants that include microsatellites may serve as efficient agents for adaptive evolution (King and Kashi 2009).

In all classes of repetitive DNA, there appears to be a general trend of increased matrix length throughout evolutionary time. Moreover, highly repetitive sequences tend to accumulate in regions of low recombination, such as centromeres and telomeres, whereas repetitive regions in euchromatin are much more susceptible to crossing-over (Pathak and Ali 2012).

Overall, the chromosome hybridization of microsatellites demonstrated contrasting patterns of abundance and localization of these sequences in the chromosomes of *Cichla monoculus*, *Pterophyllum scalare* and *Symphysodon discus*, which indicates that the repetitive sequences have accumulated differently among the genomes. Although the three species exhibited a wide distribution of microsatellites (GAATA)_8_ and (CA)_16_ in their genome, clustering of these markers was observed in *Pterophyllum scalare*, which represents a derived species in the phylogeny of Cichlinae (Smith et al. 2008). Clustering of repetitive sequences in derived species was also observed for transposable elements of this fish family (Gross et al. 2009; Valente et al. 2011; Schneider et al. 2013b). The association between microsatellites and the abundance of retrotransposable elements has been suggested as a mechanism that may increase the number of microsatellites (Nadir et al. 1996). However, in several organisms, a relationship between the high density of transposable elements and a high rate of microsatellites is not observed (Schlötterer 2000, 2015).

Position of the microsatellite sequences mapped in this study was similar to that observed for retroelements in the same species, with signals scattered throughout chromosomes and others clustered in terminal and centromeric regions (Schneider et al. 2013b). This outcome suggests that these sequences may be involved in the structural formation of the centromere and the telomere. Moreover, the microsatellites present in the centromeric and subtelomeric regions differ among cichlid species, which reinforces the importance of these sequences in the evolution of the different groups.

The centromere is an essential structure with several functional roles in the segregation of replicated chromosomes to daughter cells. These roles include genetic/epigenetic marking and the assembly of the protein complex of the kinetochore during the cell cycle, providing checkpoints to control mitosis, the release of sister-chromatid cohesion, chromosome migration to the cellular poles and cytokinesis (Santaguida and Musacchio 2009, Allshire and Karpen 2008, Perpelescu and Fukagawa 2011). Centromeres comprise long stretches of tandem repeats of satellite and microsatellite DNA, which are essential for binding with protein complexes (Verdaasdonk and Bloom 2011, Kalitsis and Choo 2012). Centromeric DNA sequences typically present high evolutionary rates and variation among species or chromosomes of the same species is common (Plohl et al. 2008). Thus, the centromeric region of *Pterophyllum scalare* is rich in (GAGAA)_12_ microsatellite sequences, but one chromosome pair does not display any hybridization signals. As well, in *Cichla monoculus* and *Symphysodon discus*, the centromere region is not rich in (GA)_12_ and (GAGAA)_12_.

Another chromosome region with a high evolutionary rate is the subterminal region. Typically, this region is composed of different classes of repetitive DNA that may help stabilize the terminal portion of the chromosomes because of the possibility of amplifying these sequences even in the absence of telomerase (Jain et al. 2010, Torres et al. 2011). In *Symphysodon discus*, the microsatellites (GT)_9_CA(GT)_7_CG(GT)_19_ and (CT)_14_GT(CT)_5_(CG)_2_(CT)_9_ are present in the subterminal region of two chromosome pairs, whereas these markers were not observed in the chromosomes of *Cichla monoculus* and *Pterophyllum scalare*. The same result was obtained for the microsatellite (AC)_7_, which was observed only in *Cichla monoculus*. The microsatellites (GAATA)_8_ and (GT)_13_ were observed in the subterminal regions of *Cichla monoculus* and *Pterophyllum scalare* but not in *Symphysodon discus*. In *Pterophyllum scalare*, a conspicuous marking of the microsatellite (GT)_13_ was observed in the terminal region of the largest chromosome pair, where the 18S ribosomal gene is located (Schneider et al. 2013a), indicating synteny between these two classes of repetitive DNA.

Still, heterochromatin of the cichlids analyzed here was located in the centromeric or pericentromeric regions in most of the chromosomes (Schneider et al. 2013). These regions show positive signals of hybridization for different microsatellites analyzed, as well as other repetitive elements (Schneider et al. 2013). The most common cellular mechanism that prevents activation and expansion of repetitive elements is the formation of heterochromatin over their sequences and three epigenetics pathways interconnected ensure the silencing of their elements: methylation of H3K9, DNA methylation and the germ-line specific PIWI pathway (Padeken et al. 2015).

The regulation of the repetitive sequences is not yet clear and depends largely on new technologies to clarify their function (Padeken et al. 2015), but the comparative mapping data presented provide novel information on the structure and organization of the repetitive region of the genome of cichlids and suggest that microsatellites contribute to chromosome structure and may have played a role in the evolution of this fish family.
